# Onset of spondyloarthritis in a patient treated with adalimumab for relapsing anterior uveitis – efficacy of secukinumab on the joint domain and on ocular inflammatory relapses: A case report

**DOI:** 10.1097/MD.0000000000031554

**Published:** 2022-11-25

**Authors:** Vincenzo Raimondo

**Affiliations:** a Rheumatology Unit, Rheumatology Hospital “Madonna dello Scoglio”, Cotronei, Italy.

**Keywords:** case report, relapsing anterior uveitis, secukinumab, spondyloarthritis

## Abstract

Adalimumab, an inhibitor of tumor necrosis factor-alpha, is a systemic non-corticosteroid agent approved for the treatment of noninfectious uveitis. Secukinumab, a human monoclonal antibody inhibiting interleukin 17A, is also being investigated as a potential treatment for noninfectious uveitis.

**Patient concerns:**

A 32-year-old Italian woman receiving adalimumab for relapsed anterior uveitis developed axial and peripheral clinical manifestations of spondyloarthritis during treatment.

**Diagnosis:**

Physical, laboratory and instrumental examination confirmed axial and peripheral spondyloarthritis associated with uveitis.

**Intervention:**

We decided to administer secukinumab 150 mg/month and interrupted the treatment with adalimumab 40 mg/2 weeks.

**Outcomes:**

The patient reported an evident remission of symptoms and improvement in clinical conditions.

**Lessons:**

Here we show the therapeutic efficacy of the switch from adalimumab to secukinumab, with remission of joint symptoms and reduction of inflammation indices, in the absence of new relapses of uveitis. This case suggests that secukinumab is primarily effective and safe on joints pain of an inflammatory nature in patients with anterior uveitis who develop spondyloarthritis as an extra-ocular symptom, while also seeming to be effective in preventing ocular symptoms recurrence.

## 1. Introduction

Anterior uveitis is an autoimmune condition which can occur as an isolated pattern (idiopathic), or associated with rheumatic or non-rheumatic conditions including spondyloarthritis, Behçet disease, inflammatory bowel disease, juvenile idiopathic arthritis, psoriasis and sarcoidosis. It represents at least 50% of the cases of noninfectious uveitis. Relapsing acute anterior uveitis, often unilateral, is the most common extra-articular manifestation in spondyloarthritis.^[1]^

Noninfectious uveitis represents the majority of uveitis occurrences (up to 90%) in the developed countries and the most common cause of this disease is HLA-B27-associated anterior uveitis (4%–32%).^[2–4]^ It is a serious sight-threatening condition whose pathogenesis has often autoimmune nature. It causes inflammation of the uveal tissues of the eye, including the iris, ciliary body, choroid, sclera, retina, retinal blood vessels, and the optic nerve. It is estimated to cause about 10% to 15% of the cases of total blindness and up to 20% of legal blindness, representing the 5th leading cause of visual loss in the developed countries.^[5]^ Uveitis can occur in all age groups and, differently from other age-related ocular diseases, adults aged between 20 and 50 are those most affected.^[6,7]^ The epidemiology of uveitis is influenced by several factors such as genetic, environmental and socioeconomic factors and this accounts for the diversity of the incidence worldwide.^[8]^

Currently, the standard treatment for noninfectious uveitis is topical and systemic steroid therapy in combination with immunosuppressants such as cyclosporine, methotrexate, sulfasalazine, mycophenolate mofetil and azathioprine. However, a significant proportion of these cases cannot be controlled only using corticosteroids and immunosuppressants. Thus, tumor necrosis factor-alpha (TNF-α) inhibitors such as adalimumab have also been approved for the treatment of noninfectious uveitis.^[9]^

Interleukin 17A (IL-17A) is a cytokine with a key role in promoting chronic inflammation and consequent tissue damage in spondyloarthritis. Secukinumab, a human monoclonal antibody inhibiting IL-17A, has been approved for the treatment of psoriasis, psoriatic arthritis and ankylosing spondylitis and it is also being investigated as a potential treatment for noninfectious uveitis.^[10]^

Here we present a case report in which secukinumab has shown efficacy on the joint domain and on ocular inflammatory relapses in a patient affected by anterior uveitis in treatment with adalimumab.

## 2. Case presentation

This is a case of a female patient diagnosed with anterior uveitis since 1998, still being followed in outpatient regimen at the Ocular Inflammatory and Autoimmune Diseases Outpatient Clinic, University Hospital of Parma. Written informed consent was obtained from the patient for the purpose of publication. Regarding family history, a paternal uncle suffering from rheumatoid arthritis was reported, while during personal physiological anamnesis emerged a spontaneous abortion and tonsillectomy. The patient has no children, no allergies or other major pathologies and was unemployed.

The patient reported many episodes of relapsing uveitis for which surgery (bilateral cataract, right vitrectomy) and pharmacological approaches were necessary.

Over the years, she underwent therapy with cyclosporine, methotrexate, hydroxychloroquine and azathioprine. The last episode of uveitis (anterior) dated back to 2014. From July 2020 she was on therapy with adalimumab and prednisone, at a dosage of 5 mg for four days a week.

The first extra-ocular manifestation occurred in 2019, characterized by arthritis of the right elbow and resolved after a short steroid cycle.

The patient came to our observation for the first time in April 2021 due to the appearance of neck and back pain, right knee pain and temporomandibular joints pain of inflammatory nature, only partially responsive to paracetamol and ketoprofen. Laboratory tests showed an increase in inflammation indices: erythrocyte sedimentation rate (ESR) 57 mm, C-reactive protein (CRP) 16 mg/L. Moreover, the clinical condition affected the psychological state of the patient who was looking for a pregnancy.

On physical examination, sharp pain and impossibility of the active and passive extension maneuver of the right elbow were observed, along with pain on palpation of the medial joint line of the right knee, which appeared swollen, limited and painful in flexion extension.

Given the clinical picture, we suspected axial and peripheral spondyloarthritis associated with uveitis. We requested the following examinations:

- hands, right elbow, cervical-dorsal-lumbar spine pelvis X-ray;
- right elbow ultrasound;
- pelvis magnetic resonance imaging (MRI);
- HLA-B27 typing.

We decided to maintain the adalimumab therapy unchanged until the outcome of the examinations, increasing the steroid (prednisone 12.5 mg/day with progressive decrease of the dosage up to 6.25 mg/day).

After 1 month (May 2021) the patient reported benefit from the increase of the steroid only on the peripheral component. The inflammatory rachialgia persisted. The bath ankylosing spondylitis disease activity index (BASDAI) was 9.

The following picture was highlighted from the exams required:

- pelvis X-ray: sclerosis of the right sacroiliac joint with preserved joint lines;
- MRI of the pelvis for sacroiliac: sacroiliitis on the right, adnexal cyst on the left;
- ultrasound and radiography of the right elbow: within normal limits;
- HLA-B27 typing: negative.

After gynecological evaluation, aimed to ascertain the nature of the adnexal cyst, phone consultation with ophthalmologists and appropriate pre-therapy screening, given the onset of sacroiliitis during treatment with anti-TNF, we decided to introduce secukinumab 150 mg/month, after the induction period. At the same time the patient interrupted the treatment with adalimumab 40 mg/2 weeks.

After one month of therapy (July 2021) the patient reported an evident improvement in clinical conditions: remission of night pain and morning stiffness. Sporadic pain in the right elbow persisted, effectively treated with nonsteroidal anti-inflammatory drugs (NSAIDs) as needed. The inflammation indices appeared negative from laboratory tests: ESR 7 mm and CRP 3.4 mg/L. She denied intercurrent infectious episodes. BASDAI was 3.2.

In September 2021, the patient informed us that she had just discovered to be pregnant. At this point, as per guidelines, we switched to certolizumab.

We saw the patient again in December 2021: the remission of the disease persisted and the pregnancy proceeded without any problems. Inflammation indices were within the physiological values (ESR 8 mm, CRP 4.3 mg/L). In June 2022, in persistent remission of the disease, the patient gave birth to a healthy girl.

## 3. Discussion

Secukinumab is a human monoclonal antibody able to inhibit IL-17A, a cytokine with a key role in promoting chronic inflammation and consequent tissue damage in spondyloarthritis. Relapsing acute anterior uveitis, often unilateral, is the most common extra-articular manifestation in spondyloarthritis, sharing the pathogenetic process with the latter. Similarly to the entheses, the ciliary body is also a highly dynamic structure subjected to mechanical stress. Mechanical stress and microbial infections determine the production of IL-23 by macrophages; this cytokine plays a key role in the differentiation of Th17 lymphocytes, a process that leads to the production of IL 17 which, as mentioned, is the cytokine responsible for inflammation and related tissue damage. Noteworthy, an increase in Th17 and IL-17A was observed in the serum of patients with acute anterior uveitis associated with positivity of HLA-B27.^[11]^ Therefore, given the selective inhibition of IL-17A, secukinumab has a potential role in the prevention of relapse of acute anterior uveitis.^[12]^ The efficacy of the drug in the treatment of noninfectious uveitis was evaluated in three randomized, double-blind, phase III, multicenter clinical trials (vs placebo). In particular, in the SHIELD study were selected patients with uveitis related to Behçet disease, while in the INSURE and ENDURE studies, patients with active and quiescent uveitis, respectively, unrelated to Behçet disease. Out of a total of 118 enrolled patients, no significant difference was observed in the number of uveitis relapses when comparing secukinumab and placebo.^[13]^ However, the INSURE and ENDURE studies ended precociously and did not take into account the fact that the different forms of uveitis do not share the same pathophysiological process.^[10]^ A study evaluating data from a Swedish registry, showed that the use of secukinumab in patients suffering from spondyloarthritis with associated anterior uveitis, predisposes to a higher risk of ocular recurrence than those patients treated with infliximab, adalimumab and certolizumab. However, this data is contradicted by a post hoc analysis carried out on data from the MEASURE 1, 2 and 3 studies, conducted on patients suffering from ankylosing spondylitis treated with secukinumab evaluated at the 156th week, where it is shown that the rate of uveitis at onset and exacerbation does not significantly differ from that observed for anti-TNFs.^[14]^

## 4. Conclusions

Given the onset of spondyloarthritis with axial and peripheral presentation in a patient treated with adalimumab for relapsing anterior uveitis, we decided to change the therapeutic strategy by inserting an IL-17A inhibitor drug. Secukinumab, besides having a key role in the therapy of spondyloarthritis, has also shown, in this case and in the above-mentioned literature, to be effective in preventing the recurrence of anterior uveitis.

## Acknowledgements

Medical writing support was provided by Antonella Managò on behalf of Health Publishing & Services Srl.

## Author contributions

**Conceptualization:** Vincenzo Raimondo.

**Investigation:** Vincenzo Raimondo.

**Supervision:** Vincenzo Raimondo.

**Writing – original draft:** Vincenzo Raimondo.
